# CAR T-cell-associated neurotoxicity in central nervous system hematologic disease: Is it still a concern?

**DOI:** 10.3389/fneur.2023.1144414

**Published:** 2023-04-06

**Authors:** Roser Velasco, Alberto Mussetti, Macarena Villagrán-García, Anna Sureda

**Affiliations:** ^1^Neuro-Oncology Unit, Department of Neurology, Hospital Universitari de Bellvitge-Institut Català d'Oncologia, Barcelona, Spain; ^2^Department of Cell Biology, Physiology and Immunology, Institute of Neurosciences, Cerdanyola del Vallés, Spain; ^3^Department of Hematology, Catalan Institute of Oncology, Hospital Duran i Reynals, Institut d'Investigació Biomèdica de Bellvitge (IDIBELL), L'Hospitalet de Llobregat, Barcelona, Spain; ^4^French Reference Center on Paraneoplastic Neurological Syndromes and Autoimmune Encephalitis, Hospices Civils de Lyon, Hôpital Neurologique, Bron. UMR MeLiS team SynatAc, INSERM1314/CNRS5284, Lyon, France; ^5^Medicine Department, Universitat de Barcelona, Barcelona, Spain

**Keywords:** CAR T-cell therapy, CNS relapse, ICANS, CNS infiltration, leukemia, lymphoma, myeloma, neurotoxicity

## Abstract

Chimeric antigen receptor (CAR) T-cell systemic immunotherapy has revolutionized how clinicians treat several refractory and relapsed hematologic malignancies. Due to its peculiar mechanism of action, CAR T-cell-based therapy has enlarged the spectrum of neurological toxicities. CAR T-cell-associated neurotoxicity—initially defined as CAR T-cell-related encephalopathy syndrome (CRES) and currently coined within the acronym ICANS (immune effector cell-associated neurotoxicity syndrome)—is perhaps the most concerning toxicity of CAR T-cell therapy. Importantly, hematologic malignancies (especially lymphoid malignancies) may originate in or spread to the central nervous system (CNS) in the form of parenchymal and/or meningeal disease. Due to the emergence of deadly and neurological adverse events, such as fatal brain edema in some patients included in early CAR T-cell trials, safety concerns for those with CNS primary or secondary infiltration arose and contributed to the routine exclusion of individuals with pre-existing or active CNS involvement from pivotal trials. However, based primarily on the lack of evidence, it remains unknown whether CNS involvement increases the risk and/or severity of CAR T-cell-related neurotoxicity. Given the limited treatment options available for patients once they relapse with CNS involvement, it is of high interest to explore the role of novel clinical strategies including CAR T cells to treat leukemias/lymphomas and myeloma with CNS involvement. The purpose of this review was to summarize currently available neurological safety data of CAR T-cell-based immunotherapy from the clinical trials and real-world experiences in adult patients with CNS disease due to lymphoma, leukemia, or myeloma. Increasing evidence supports that CNS involvement in hematologic disease should no longer be considered *per se* as an absolute contraindication to CAR T-cell-based therapy. While the incidence may be high, severity does not appear to be impacted significantly by pre-existing CNS status. Close monitoring by trained neurologists is recommended.

## 1. Introduction

Commercially available chimeric antigen receptor (CAR) T-cell systemic immunotherapy includes administering a single infusion of a CAR T-cell product that had been engineered by the transduction of the CAR gene into healthy, autologous T cells using a viral vector. CAR T cells are expanded *ex vivo* and infused back into the patient after lymphodepletive chemotherapy has been performed. These modified T cells bind to target antigen-positive cells, resulting in activation and *in vivo* proliferation of CAR T cells to begin eliminating target malignant, antigen-positive cells. Since 2017, six CAR T-cell-based therapies have been approved by the Food and Drug Administration (FDA) to treat hematologic cancers, including (in alphabetical order) Abecma (idecabtagene vicleucel, ide-cel), Breyanzi (lisocabtagene maraleucel, liso-cel), Carvykti (ciltacabtagene autoleucel, cilta-cel), Kymriah (tisagenlecleucel, tisa-cel), Tecartus (brexucabtagene autoleucel, brextu-cel), and Yescarta (axicabtagene ciloleucel, axi-cel). CD19 is a cell surface antigen that is expressed on malignant and normal B-cells. CD19-directed CAR T-cell-based therapy has been successful in treating several B-cell lineage malignancies, including systemic diffuse large B-cell lymphoma (DLBCL), B-cell acute lymphoblastic leukemia (B-ALL), mantle cell lymphoma (ML), and follicular lymphoma (FL). B-cell maturation antigen (BCMA) is a member of the tumor necrosis factor receptor superfamily, which is highly expressed in multiple myeloma (MM) cells. BCMA CAR T-cell-targeted therapy is approved for refractory/relapsed MM (Table 1).

**Table 1:** CAR T-cell-based therapies approved to treat hematologic cancers.

**Generic name**	**Brand name**	**Target antigen**	**Hematologic disease**	**Population**	**FDA approval trial (references)**	**ICANS rate (grade 3–4)**
Tisagenlecleucel (tisa-cel)	Kymriah	CD19	R/R B-ALL	Children and young	NCT02435849 ELIANA trial (4)	40% (13%)
			R/R B-NHL	Adult	NCT02445248 JULIET trial (5)	21% (12%)
			R/R Follicular Lymphoma	Adult	NCT03568461 ELARA trial (6)	37.1% (>3.3%)
Axicabtagene ciloleucel (axi-cel)	Yescarta	CD19	R/R B-NHL	Adult	NCT02348216 ZUMA-1 trial (7)	64% (28%)
			R/R Follicular Lymphoma	Adult	NCT03105336 ZUMA-5 trial (8)	59% (19%)
Brexucabtagene autoleucel (brexu-cel)	Tecartus	CD19	R/R B-ALL	Adult	NCT02614066 ZUMA 3 (9)	60% (25%)
			R/R MCL	Adult	NCT02601313 ZUMA 2 (10)	63% (31%)
Lisocabtagene maraleucel (liso-cel)	Breyanzi	CD19	R/R B-NHL	Adult	NCT02631044 TRANSCEND NHL 001 (11)	30% (10%)
Idecabtagene vicleucel (ide-cel)	Abecma	BCMA	R/R MM	Adult	NCT03361748 KARMMA trial (11)	18% (3%)
Ciltacabtagene autoleucel (cilta-cel)	Carvykti	BCMA	R/R MM	Adult	NCT03548207 CARTITUDE-1 (12)	21% (9%)

ICANS, immune effector cell-associated neurotoxicity syndrome; RR, relapsed/refractory; ALL, acute lymphoblastic leukemia; NHL, non-Hodgkin lymphoma; MCL, mantle cell lymphoma; MM, multiple myeloma. ^*^Per CTCAE v4.03.

Treatment-related neurotoxicity due to cancer therapy in hematologic disease is not new. Both chemotherapy (systemic and intrathecal) and radiotherapy can cause neurological side effects in both the CNS and peripheral nervous system (PNS). Some CNS complications may appear during treatment, while others will present months or even years later. Classical neurotoxicity caused by traditional anticancer therapies is well-known and widely described (1, 2). It should be noted that CAR T-cell-based therapy has dramatically changed the landscape of cancer therapy and widened the range of neurological toxicities with the emergence of CAR T-cell-associated neurotoxicity. Initially defined as CAR T-cell-related encephalopathy syndrome (CRES) and currently coined within the acronym ICANS (immune effector cell-associated neurotoxicity syndrome), it is a severe complication of CAR T-cell-based therapy due to its potentially life-threatening nature.

**Figure 1:**
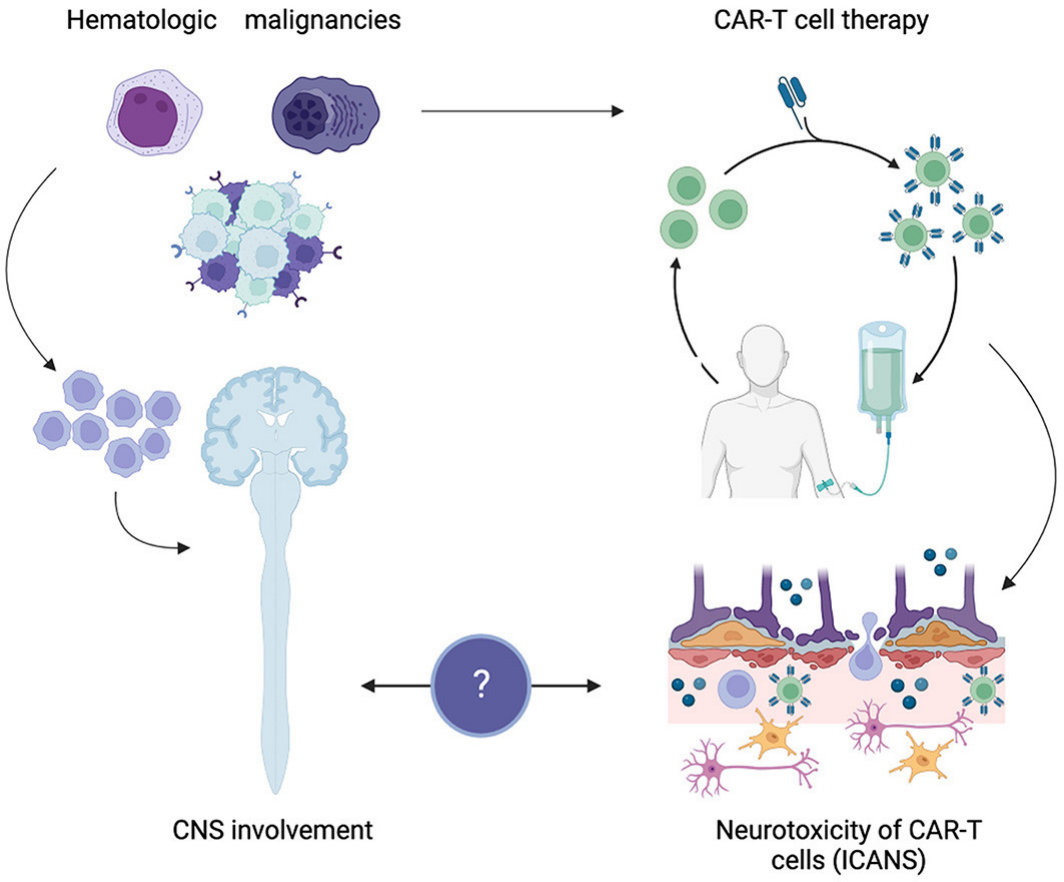
CAR T-cell-associated neurotoxicity in central nervous system hematologic disease. Created with BioRender.com.

Furthermore, CNS involvement—defined by the presence of malignant cells that have invaded the CNS—can be seen in lymphoma (both primary and secondary), lymphoblastic leukemia, and, on rare occasions, in MM. However, the safety of CAR T-cell-based therapy in CNS involvement due to hematologic disease has not yet been well-characterized, given the routine exclusion of these patients from trials, partially related to the fear that it could exacerbate any potential neurotoxicity associated with CAR T cells. To date, whether CNS involvement raises the risk and/or severity of CAR T-cell-related neurotoxicity is unknown. It remains a relative contraindication in the recent guidelines (3). The aim of this brief review is to update the knowledge gap by summarizing currently available neurological safety data of CAR T-cell-based immunotherapy from the latest clinical trials and real-world experiences in patients with CNS disease due to lymphoma, leukemia, or myeloma (Figure 1). Despite reports of promising outcomes with CAR T-cell-based therapy in primary and secondary CNS hematologic disease, the antitumor efficacy of such immunotherapy does not fall within the scope of the present review and will not be discussed.

## 2. CAR T-cell-associated neurotoxicity

### 2.1. Frequency

The rate of incidence and severity of ICANS will vary widely depending on the host and product. Table 1 summarizes the registrational CAR T-cell trials already approved by the FDA and the rate of ICANS observed. Importantly, most CAR T-cell trials have traditionally excluded patients with prior or active CNS involvement due to a concern for increased neurotoxicity. In pivotal trials such as TRANSCEND NHL 001, 2.6% of all enrolled patients had CNS disease. In the ELIANA trial, 13.9% of patients had a history of CNS disease.

Overall, ICANS usually affects 20–70% of patients treated with CD19 CAR T cells in B-NHL or B-ALL (3, 13, 14), with higher rates of neurotoxicity associated with the use of the CD28 costimulatory domain (axi-cel) in comparison with the CD4-1 BB costimulatory domain (tisa-cel or liso-cel) (15). A recently published systematic review including 23 observational studies and a total of 1,666 adult patients who received CAR T-cell-based therapy to manage hematologic malignancies reported a highly variable ICANS incidence of 37.5–77% (16). This type of neurotoxicity may be severe and occasionally fatal (16). Severe ICANS (grades 3–4 according to NCI-CTC grading scales) may occur in up to 31% of patients receiving anti-CD19 CAR T cells and 9 % of patients with MM receiving anti-BCMA CAR T-cell therapy (3). Recently, five ICANS-related deaths across two studies enrolling a total of 233 participants were reported (16). With BCMA CAR T-cell-approved drugs, overall, CAR T-cell-associated neurotoxicity is lower: It has been reported in up to 21% of patients with MM. In this population, the combination of anti-BCMA and anti-CD19 CAR T cells—still not FDA-approved—has shown a low rate of neurotoxic events (11%), with 3% with grade ≥3 (17).

### 2.2. Clinical presentation

Typically, ICANS is a type of toxic encephalopathy, predominantly manifesting as frontal encephalopathy (14, 18). However, the spectrum of neurological manifestations of CAR T-cell-associated neurotoxicity can highly vary in type and severity (16). Following CAR T-cell transfusion, the common initial ICANS symptoms include dysgraphia, word-finding difficulties, tremors, confusion, and somnolence (19). Hesitant speech and deterioration in handwriting are prominent and can rapidly progress to expressive aphasia and mutism (18, 20). Furthermore, patients may develop a wide range of clinical manifestations like seizures, headaches, focal deficits, and even a decreased level of consciousness. In worse clinical scenarios, intracranial hypertension may occur and cause coma (16). ASTCT published guidelines for ICANS consensus grading based on the immune effector cell-associated encephalopathy (ICE) score, depressed level of consciousness, seizure, motor findings, and cerebral edema (1, 21).

ICANS will usually develop early within the 1st week of a CAR T-cell infusion. Its duration will be limited in most patients. The pooled mean onset and duration of ICANS in a systematic review were 6.4 ± 3.2 days and 8.3 ± 10.5 days, respectively (16). However, 10% of patients develop “delayed ICANS” >3 weeks after the infusion following CRS remission (3). It should be noted that fluctuating symptoms with intermittent symptom-free episodes—even in high-grade neurotoxicity—have been described (18, 22). In severe cases, ICANS can progress in a few hours (18) and results in quick, fatal outcomes in a minority of patients due to fulminant cerebral edema (1–2% estimated incidence) (23, 24), especially in CD28 costimulatory domain constructs. Although ICANS is usually mild in severity and reversible, some patients will develop a more severe and even life-threatening form of the syndrome. Fatal neurotoxicity has been described in pivotal studies [JCAR-015 (NCT02535364)], prompting even trial cessation, with an overall incidence of 3–9% after CD19-directed CAR T-cell infusion (16, 22, 25, 26).

Other neurotoxicity events that do not fit the current definition for ICANS have also been described with CAR T-cell-based therapies, especially in patients with MM. In the CARTITUDE-1 trial, 5% of patients with MM reported movement and neurocognitive treatment-emergent adverse events (MNTs) with cilta-cel, comprising a cluster of movement (e.g., micrographia and tremors), cognitive changes (e.g., memory loss and disturbances in attention), and personality changes (e.g., reduced facial expression and flat affect) (3). These neurological symptoms and signs were not temporally associated with CRS, presenting a later onset (median day 27) and longer time to resolution (median 75 days) (3, 11, 27). Increasing fatigue can be the first complaint, and clinical syndrome with features of parkinsonism has been seen in patients with high-grade neurotoxicity. These late-onset parkinsonian symptoms in these patients seem to occur as a result of damage to the basal ganglia. Neuropathology findings from autopsied patients showed focal basal ganglia lymphocytic infiltration but intact substantia nigra (27, 28). In addition, a lack of response to treatment with levodopa and the negative dopamine uptake (27, 28) and decreased uptake in the caudate nucleus in FDG-PET/CT (28) would support a distinct pathophysiology from Parkinson's disease.

### 2.3. Pathophysiology and management

The mechanism of CAR T-cell neurotoxicity remains to be well-elucidated, and several mechanisms of ICANS after CAR T-cell treatment have been described (29). The most widely accepted mechanism of CAR T-cell neurotoxicity is driven by systemic inflammation and cytokine production. Indeed, ICANS usually overlaps and correlates with CRS, but it has also been occasionally reported to occur independently from CRS (30). Several risk factors related to CAR T-cell neurotoxicity have been described, such as pre-treatment disease burden, *in vivo* CAR T-cell expansion, early and severe CRS, and CAR T-cell dose (3). In fact, faster CAR T-cell expansion *in vivo* has been related to the onset and severity of ICANS (19, 22, 25, 31).

There is strong evidence that highlights the role of endothelial activation during the development of ICANS (25, 30). It occurs shortly after CAR T-cell administration yet precedes ICANS, inducing systemic capillary leak and subsequent dysfunction of the blood–brain barrier (BBB). Increased permeability of BBB facilitates infiltration of peripheral immunocytes, CAR T cells, and cytokines into the CNS. This triggers a further local inflammatory response and CNS inflammation with subsequent astrocyte injury and microglial activation. It then results in abnormal neuronal function related to the dysregulation of neuroglial cells and neurotransmitters (29). In addition, cerebral multifocal hemorrhage in patients with ICANS indicates thrombotic microangiopathy compromising BBB function (29). Targeting of cerebral CD19-expressing pericytes can disrupt the BBB and contribute to the development of ICANS. However, ICANS can occur with CAR T-cell therapies targeting CD20, CD22, BCMA, and CD19, making it unlikely for the occurrence of such a syndrome to be solely caused by the nature of the target antigen (30). Contradictory results regarding BCMA expression in CNS have been reported (28, 32, 33).

In addition, myeloid cell hyperactivation contributes significantly to the development of ICANS. Myeloid cell-derived cytokines, notably IL-1 and IL-6, have been shown to drive systemic inflammation, correlating with severe ICANS development (30, 34). Importantly, CAR T cells can migrate and persist in CSF (35–38) regardless of whether systemic or even CNS disease is present (39). However, it is unknown whether the number of activated CAR T cells inside the CNS is related to ICANS (30). Some studies fail to identify this association (40), and other studies support that the neurotoxicity grade correlates with CSF cytokine levels and not with CSF CAR T-cell levels (14).

ICANS management is done according to the ASCTC guidelines. Supportive care alone can be used in very mild (grade 1) cases, but corticosteroids, like dexamethasone or methylprednisolone, are the mainstays of ICANS treatment. Initially, dexamethasone i.v. 10 mg/6 h for 1–3 days should be prescribed. In the absence of improvement or deterioration, dexamethasone i.v. 20 mg/6 h for 3 days and progressive tapering within 3–7 days should be considered. In severe or refractory cases, escalation to methylprednisolone i.v. 1,000 mg/day for 3 days and further progressive tapering is recommended (3). No specific guidelines exist for the treatment of steroid-refractory ICANS (3). Based on prior studies, using tocilizumab—a monoclonal antibody that cannot cross the BBB and blocks IL-6R in peripheral tissues—to manage ICANS in the absence of concurrent CRS is not currently recommended. This is due to concerns about a possible worsening of ICANS in tocilizumab's ability to raise CSF IL-6 levels (16). Useful agents adjunct to steroids in the management of steroid-refractory ICANS include Anakinra, an IL-1R antagonist, with no immediate effect in improving neurological symptoms. It is considered to modestly shorten the duration of neurological toxicities due to improvements in clinical and laboratory indices of inflammation (41, 42). Finally, intrathecal treatment with cortisone, cytarabine, and methotrexate can serve as a possibility in steroid-refractory ICANS (3).

## 3. CNS involvement in lymphoma, leukemia, and myeloma

### 3.1. Lymphoma

CNS lymphoma is a form of extranodal non-Hodgkin lymphoma (NHL) that primarily (PCNSL, primary central nervous system lymphoma) affects the brain, spinal cord, meninges, and/or eyes. It can also be secondary (SCNSL, secondary central nervous system lymphoma) to systemic lymphoma. Most (>90%) PCNSLs are diffuse large B-cell malignancies in origin (43).

**Figure 2:**
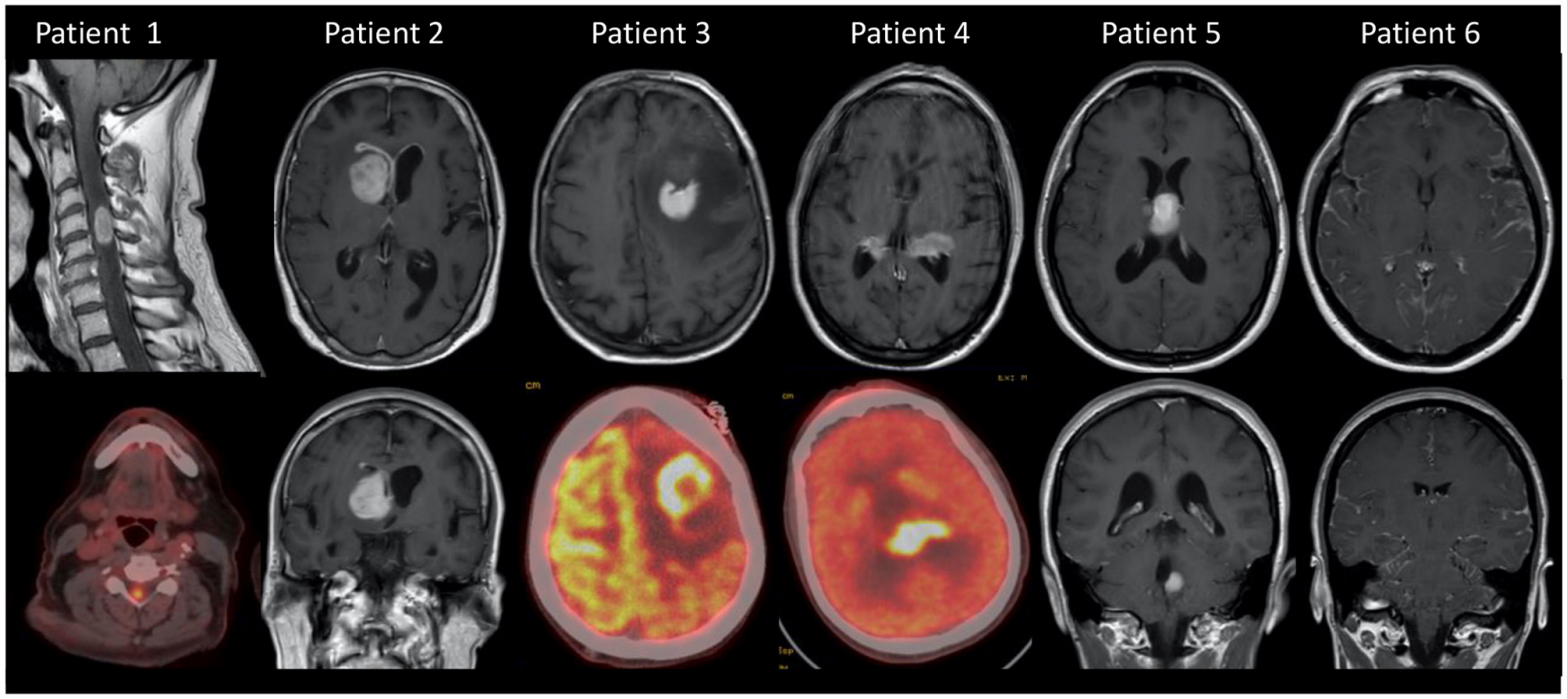
MRI or PET scan images of patients with hematologic CNS disease. Patient 1: SCNSL with spinal cord involvement. Patient 2: SCNSL relapse with leptomeningeal involvement. Patient 3: SCNSL at the frontal left lobe. Patient 4: PCNSL. Patient 5: Multifocal PCNSL. Patient 6: Leptomeningeal diffuse infiltration in a patient with leukemia.

SCNSL is more likely to occur in a relapsed setting of a systemic NHL. Secondary CNS relapses are uncommon yet with devastating complications; incidence can reach up to 15% in high-risk patients (44). Elevated risk factors of CNS relapse in the validated 6-score risk model (CNS-IPI) include age >60, performance status >1, elevated LDH, extranodal sites >1, stage III or IV disease, and kidney or adrenal involvement (45). Double-hit lymphoma is also associated with a higher risk of CNS relapse (44). CNS recurrence is typically leptomeningeal or confined to the brain parenchyma rather than the spinal cord (Figure 2). Mantle cell lymphoma (MCL) comprises ~3% of adult NHL cases, and CNS involvement has an incidence of 4%. CNS relapse in MCL is typically leptomeningeal rather than parenchymal (46). In addition to known IPI risk factors, Ki-67 ≥30% was the strongest risk factor predicting CNS relapse, with a 2-year cumulative incidence of 25.4% (47).

### 3.2. Acute lymphoblastic leukemia

CNS involvement in acute leukemias remains underdiagnosed. It is present in ~5% of adults with CNS leukemia at diagnosis and has a shorter overall survival in comparison with patients without CNS involvement. CNS involvement is detected in up to 30–40% of patients at relapse due to CNS tropism of ALL blasts (48). The known risk factors for CNS involvement in ALL include mature B-cell or T-cell phenotype, younger patient age, extramedullary disease (EMD), elevated LDH and white blood cell count at diagnosis, and Philadelphia chromosome positivity (49) (Figure 2).

### 3.3. Myeloma

**Figure 3:**
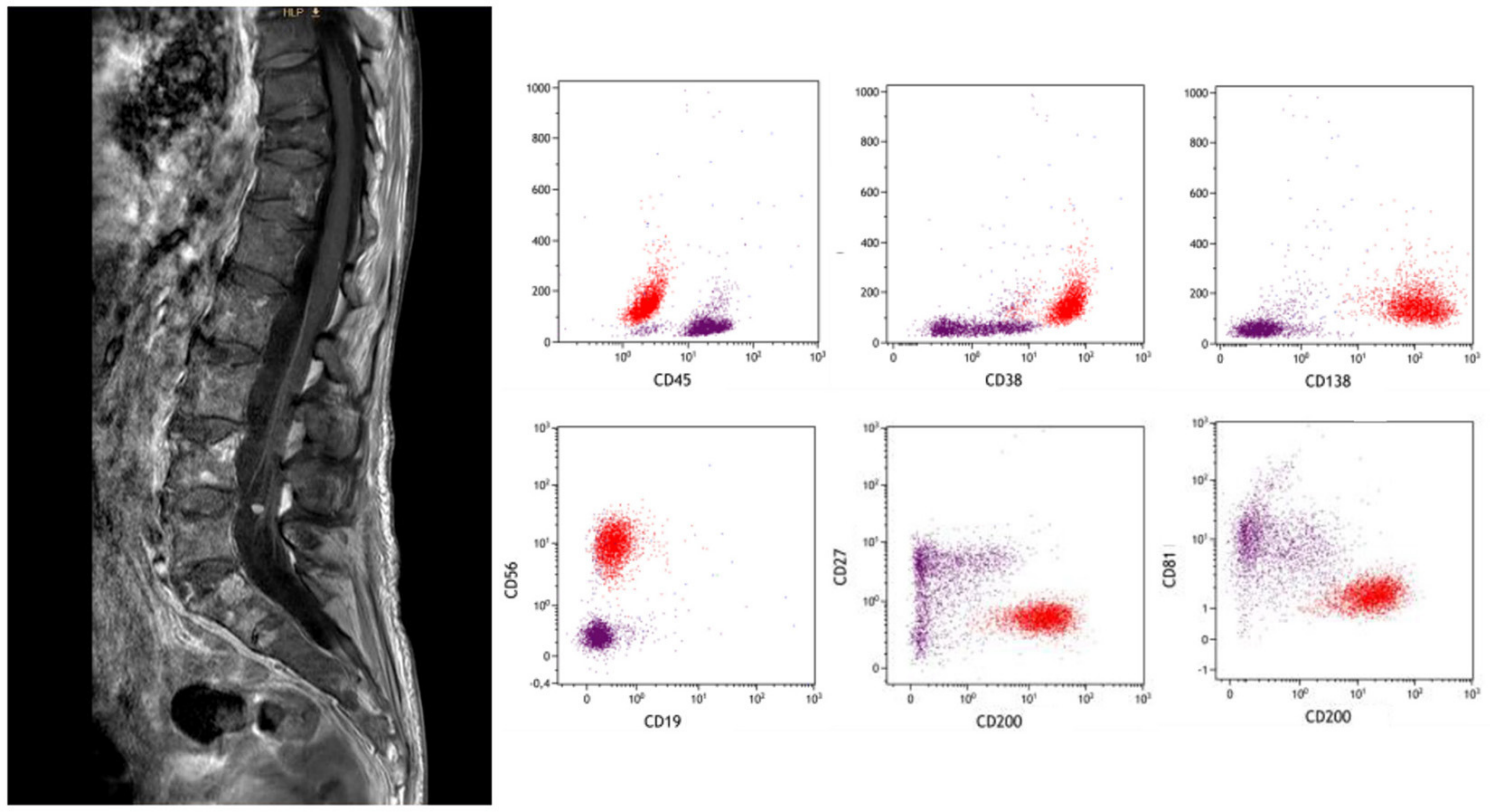
A 67-year-old woman with leptomeningeal MM presented with rapidly progressive paraparesis. Linear enhancement along the conus and cauda equina nerve roots (left panel, denoted by arrows). Subtle nodularity of the ventral L4 nerve root. CSF flow cytometry was positive for myelomatous involvement (in red).

MM is a hematologic malignancy, in which a proliferation of clonal plasma cells (PCs) occurs within the bone marrow. Extramedullary involvement (or EMD) represents an aggressive form of MM, characterized by the ability of a clone and/or subclone to thrive and grow independently of the bone marrow microenvironment. EMD is thought to be related to hematogenous spread when myeloma cells show decreased cell surface receptor expression and allow cells to escape from the bone marrow (50). Among them, the CNS localization of MM accounts for about 1% of all MM during the disease course; it is even rarer at diagnosis. Extramedullary localizations in CNS include intraparenchymal, pachymeningeal, and/or leptomeningeal. In the spinal canal, extramedullary myeloma usually manifests as an epidural soft tissue mass that can be contiguous or non-contiguous with the bone. CNS involvement prognosis is even more dismal than for EMD in other locations particularly if there is leptomeningeal involvement (51) (Figure 3).

## 4. ICANS in patients with hematologic malignancies and CNS involvement

### 4.1. Frequency

Increasing individual or short-series data related to CAR T-cell safety and ICANS in patients with CNS disease have been reported in patients with PCNSL (52), SCNSL (19, 35, 53–56), intravascular lymphoma (57, 58), MCL (59), leukemia (39, 60–62), and myeloma (63–66). Table 2 summarizes larger studies.

**Table 2:** Studies including ≥5 patients with CNS disease and reported data on ICANS.

**References**	**Type of study**	**Type of CAR-T**	***N***	**CNS disease (localization)**	**ICANS Severity *n*/total (%)**
Abramson et al. (11)	Clinical trial	Liso-cel	7	SCNSL	2/7 (29 %) 2/2 (100 %)
Ahmed et al. (67)	Single-center retrospective	Axi-cel (3) Tisa-cel (4)	7	SCNSL 5 parenchyma/2 lepto	3/7 (42.8 %) 1/3 (33.3%)
Alcantara et al. (68)	Multicenter retrospective	Axi-cel (2) Tisa-cel (7)	9	PCNSL 8 parenchyma/1 lepto	5/9 (55.5%) 2/5 (40%)
Ayuk et al. (69)	Multicenter retrospective	Axi-cel (14) Tisa-cel (14)	28	SCNSL 16 parenchyma/6 lepto/6 both	13/28 (46%) 4/13 (31%)
Bennani et al. (70)	Multicenter retrospective	Axi-cel	15	SCNSL 4 parenchyma/10 lepto/1 NA	13/15 (87%) 5/13 (38.4%)
Frigault et al. (71)	Single-center retrospective	Tisa-cel	8	SCNSL 3 parenchyma/3 lepto/2 both	3/8 (37.5%) 0/3 (0%)
Frigault et al. (36)	Phase I/II trial	Tisa-cel	12	PCNSL 10 parenchyma/2 lepto	6/12 (50%) 1/6 (16.6%)
Ghafouri et al. (72)	Single-center retrospective	Axi- or Tisa-cel	7	SCNSL 7 parenchyma/2 lepto	4/7 (57%) 2/4 (50%)
Jacobson et al. (73)	Pilot trial	Axi-cel	8^*^	6 PCNSL/3 SCNSL 8 parenchymal only	4/8 (50%) 3/4 (75%)
Jacoby et al. (74)	Multicenter retrospective	CD-19	55	ALL 16/55 active CNS	21/55 (38%) 6/21 (28.5%)
Karschnia et al. (75)	Single-center retrospective	CD19	10	SCNSL 7 parenchyma/3 lepto	6/10 (60%) 3/6 (50%)
Leahy et al. (76)	*Post-hoc* analysis	CD19	66	B-ALL	38/66 (57%) 8/38 (21%)
Li et al. (77)	Phase I trial	CD19 + CD22	5	1 PCNSL/4 SCNSL 4 parenchymal/1 both	2/5 (40%) 1/2 (50%)
Liu et al. (78)	Single-center prospective	CD19 or CD20	7	1 PCNSL/6 SCNSL 5 parenchymal	0/7 (0%)
Liu et al. (79)	Phase I trial	CD19-CD22	5^*^	Burkitt (5 evaluable safety)	5/5 (100%) 3/5 (60 %)
Nastoupil et al. (80)	Multicenter retrospective	Axi-cel	21	SCNSL History of CNS disease	NA
Qi et al. (40)	Multicenter retrospective	CD19	48	B-ALL	18/48 (37.5%) 11/18 (61.1%)
Shalabi et al. (37)	Phase I trial	CD19/CD28	13	B-cell leukemia 1 parenchymal	5/13 (38.4%) 1/5 (20%)
Siddiqi et al. (38)	Retrospective phase I	CD19	5	PCNSL	5/5 (100%) 1/5 (20%)
Sylvain et al. (81)	Multicenter retrospective	Axi-cel (4) Tisa-cel (12)	17	PCNSL 13 parenchyma/4 lepto only	9/17 (52.9%) 3/9 (33.3%)
Strati et al. (82)	Single-center retrospective	Axi-cel	8	SCNSL Prior CNS lymphoma	8/8 (100%) 5/8 (62.5%)
Tan et al. (83)	Single-center retrospective	CD19	12	B-ALL 1 parenchyma	10/12 (83%) 4/10 (40%)
Wu et al. (84)	Single-center trial	CD19/22	13	4 PCNSL/9 SCNSL	3/13 (27%) 1/3 (33.3%)
Xue et al. (85)	Single-center retrospective	CD19/20/22	17	2 PCNSL/15 SCNSL 13 parenchyma/7 lepto	6/17 (35%) 5/6 (83.3 %)
Yuen et al. (86)	Multicenter retrospective	Axi-cel	14	SCNSL (7/14 active) (3 HIV+)	6/14 (43%) 4/6 (66.66%)
Zhang et al. (87)	Multicenter retrospective	CD19/20/22	15	SCNSL 10 parenchyma/6 lepto	3/15 (20%) 1/3 (33.3%)

SCNSL, secondary central nervous system lymphoma; PCNSL, primary central nervous system lymphoma; B-ALL, B-cell acute lymphoblastic leukemia; NA, not available; HIV, human immunodeficiency virus. ^*^Patients evaluable for safety.

The occurrence and severity of CAR T-cell therapy-related neurotoxicity have been evaluated in a limited number of patients diagnosed with CNS involvement. The pooled analysis of the available data in the literature including patients with lymphoma has been recently reported in four studies. Investigators Wu et al. were the first to review the safety of CAR T cells in patients with lymphoma and secondary CNS involvement. Their research including 10 studies involving 44 patients with lymphoma concluded that patients with secondary CNS lymphoma treated with CAR T-cell products presented similar rates for any grade of ICANS (Axi-cel in CNS cohort: 56 vs. 64% in ZUMA-1; Tisa-cel in the CNS cohort: 50 vs. 21% in JULIET); grade ≥3 ICANS [Axi-cel in the CNS cohort: 39 vs. 28% in ZUMA-1; Tisa-cel in the CNS cohort: 0 vs. 12% in JULIET] (84). More recently, Yi et al. summarized the results of 11 studies reporting 58 cases of B-NHL that involved patients with PCNSL or SCNSL. Among these, 25 were assessable for ICANS. They identified that 44% (11/25) developed ICANS, and 35% presented a severe form of the syndrome (88). Furthermore, Asghar et al. reported collective data from 14 studies that comprised eight retrospective studies and six single-arm prospective studies/clinical trials, with a total of 137 patients. The purpose of the study was to evaluate the efficacy and safety of CAR T-cell therapy in primary and secondary relapsed/refractory CNS lymphoma. They identified that ICANS with a grade 3 or higher was present in 22% of the patients (*n* = 30) (89). Finally, Cook et al. performed a large, retrospective systematic review and meta-analysis including 128 patients with relapsed/refractory PCNSL (30) and SCNSL (98). According to their results, half of these patients developed ICANS. Among patients with PCNSL, 53% had ICANS of any grade (18% grade 3–4). The SCNSL cohort showed a rate of 48% for ICANS of any grade (26% had grades 3–4) (90).

It should be noted that in those studies where safety profiles were compared between CNS and non-CNS cohorts, no differences in the incidence of ICANS (11, 69, 76) nor the severity of neurotoxicity (70, 79) in patients with CNS involvement when compared to those without were seen in the majority. In contrast, a pooled *post-hoc* analysis of five clinical trials for children and young adults treated with CD19 CAR T cells for ALL at the Children's Hospital of Philadelphia recently analyzed patients who had a history of CNS involvement within the 12 months prior to CAR T-cell infusion. Patients with a history of CNS positivity 1 year before the infusion had more neurotoxic events compared to patients without a history of CNS disease (76).

With respect to clinical presentation after CAR T-cell infusion, the median time to ICANS was similar to that reported in patients without CNS involvement. Reported medians were between 3 and 8 days—mostly 5 days—and ranged from 1 to 21 days. The duration of neurotoxicity was usually short, with median rates between 3 and 14 days (ranging from 1 to 70) (36, 68, 69, 75, 76, 83, 85). The reversibility of neurotoxic events and the full recovery of ICANS under intensive management were reported in many series (9, 40, 62, 67, 72, 77, 85, 91).

It should be noted that neurological symptoms caused by specific disease infiltration into the CNS can present the same symptomatology observed with ICANS. Difficulties in differentiating ICANS from disease progression *via* imaging have been reported (36). Furthermore, the cases of pseudo-progression perhaps linked to CAR T cells have been described (68, 75).

### 4.3. CNS disease as a risk factor for ICANS

Although most studies are not powered to stratify risk by individual and pre-existing neurological comorbidity, such comorbidities have been significantly associated with an increased risk of high-grade neurotoxicity (25). This also includes some patients with CNS disease (76). Interestingly, CNS-directed radiotherapy as bridging therapy before CAR T-cell infusion was not found to be associated with a higher risk of ICANS (67, 75).

Earlier studies have shown that neurotoxicity is associated with pre-infusion disease burden and *in vivo* CAR T-cell expansion. The issue of whether parenchymal lesions increase the risk of neurotoxicity was recently explored. No difference in the type of CNS manifestation (leptomeningeal or parenchymal) was identified (10, 69). Importantly, the variable status of CNS disease can be observed in studies (86). Patients did not necessarily have active CNS involvement at the time of inclusion or leukapheresis (40, 70). In patients with leukemia, some studies suggest that high-burden CNSL before CAR T-cell infusion may predispose patients to severe neurotoxicity (83). Qi et al. identified that neurotoxic events of grades 3 to 4, which developed in 11 patients (22.9%), were associated with a higher pre-infusion disease burden in CNS (40). In the larger series of patients with leukemia, the multivariate analysis showed that patients with CNS disease were 3.42 times more likely to develop symptoms of neurotoxicity than those with no history of CNS disease. In this study, patients with CNS disease were more likely to have neurotoxicity than those in the CNS negative group; however, neurological events of grades 1 and 2 were the largest contributor to this difference. There was no increased risk of severe neurotoxicity (76). Conversely, in the multivariate analysis, CNS status at lymphodepletion in patients with CNS relapse of ALL was not significant with respect to the higher incidence of ICANS.

## 5. Conclusion

The management of central nervous system involvement in hematologic diseases remains an area of unmet medical attention. Previous reports of significant neurotoxicity related to CAR T-cell infusion led to clinicians weighing great caution when treating diseases with CNS involvement. However, to date, ICANS has not been consistently associated with CNS disease, especially in CNS lymphoma. In most series, no elevated ICANS incidence or lethal neurotoxicity occurred, and usually, ICANS symptoms were reversible. Furthermore, very few treatment-related deaths were reported. Therefore, neurotoxicity appears similar to previous reports on patients with lymphoma without CNS involvement. Despite the limitations of available data, which are mostly (>65%) retrospective in nature, obtained from mixed patients with a history of active secondary CNS disease and from small series, ICANS outcomes seen in real-world settings are fairly similar to safety data reported in trials. Ongoing trials evaluating the use of CAR T-cell therapy in CNSL (NCT04134117, NCT04608487, NCT04464200, NCT04532203, NCT02153580, and NCT03484702) will help to elucidate other questions like the impact of simultaneous CNS and systemic involvement and the difference in CAR properties in this population. In summary, growing evidence suggests that the presence of CNS disease may not be associated with a highly elevated incidence or severity of ICANS. This, in turn, supports the feasibility of CAR T-cell therapy in patients with CNS involvement and reiterates the manageability of toxicities. We believe that having CNS disease should not preclude someone from receiving CAR T-cell therapy as a treatment option due to concerns about ICANS. Although current data could indicate no clear excess neurotoxicity in this setting, vigilant close monitoring by trained neurologists is recommended for patients treated with currently available and new CAR T cell products.

## Author contributions

RV and AM conceptualized and designed the study. RV, AM, and MV-G contributed to the manuscript writing. All authors made substantial contributions to the design, acquisition of data, and analysis and interpretation of data. All authors performed the research study, acquisition, analysis and interpretation of data, and revised the manuscript for important intellectual content. All authors contributed to the article and approved the submitted version.
